# Variation in Plant Response to Herbivory Underscored by Functional Traits

**DOI:** 10.1371/journal.pone.0166714

**Published:** 2016-12-09

**Authors:** Aspen T. Reese, Gregory M. Ames, Justin P. Wright

**Affiliations:** Department of Biology, Duke University, Durham, NC, United States of America; Portland State University, UNITED STATES

## Abstract

The effects of herbivory can shape plant communities and evolution. However, the many forms of herbivory costs and the wide variation in herbivory pressure, including across latitudinal gradients, can make predicting the effects of herbivory on different plant species difficult. Functional trait approaches may aid in contextualizing and standardizing the assessment of herbivory impacts. Here we assessed the response of 26 old-field plant species to simulated defoliation in a greenhouse setting by measuring whole plant and leaf level traits in control and treated individuals. Simulated defoliation had no significant effects on any plant traits measured. However, the baseline leaf level traits of healthy plants consistently predicted the log response ratio for these species whole plant response to defoliation. The latitudinal mid-point of species’ distributions was also significantly correlated with aboveground biomass and total leaf area responses, with plants with a more northern distribution being more negatively impacted by treatment. These results indicate that even in the absence of significant overall impacts, functional traits may aid in predicting variability in plant responses to defoliation and in identifying the underlying limitations driving those responses.

## Introduction

Herbivory is a key process that shapes plant communities[1–3] and the evolution of plants[4, 5]. However, developing models to predict the effects of herbivory on plant community structure have proven difficult as plants can respond to herbivory through a complex array of pathways, each with different cost, from reducing leaf area to changes in carbon fixation and photosynthesis[6, 7]. In addition, rates of herbivory can vary greatly, thereby resulting in different selective pressure and in turn varying plant responses to the same herbivore load[8]. Functional traits provide a means of comparing species that is independent of species identity and comparable across taxa[9]. This approach can be particularly useful when researching the ecology of complex phenomena that involve many costs, such as response to herbivory.

Herbivory incurs three varieties of cost on an individual plant–lost tissue, construction, and opportunity costs–and all can be approximated or integrated into a measure of plant response using functional traits. Furthermore, a plant’s response to herbivory can further be categorized as either a tolerance or resistance strategy. The direct cost of herbivory to a plant is loss of tissue, but the impact of that loss depends on plant growth strategy and relative resource availability (e.g. Resource Availability Hypothesis[10]), because these factors shape the construction cost of new tissue and the opportunity cost of lost tissue[11, 12]. Tolerance is the ability to compensate for the fitness cost of herbivory and is often characterized by having many, cheap leaves and higher growth rates, especially under high nutrient availability[11, 13, 14]. Commonly measured functional traits, such as specific leaf area (SLA) and leaf dry matter content (LDMC) can be used as proxies for the costliness of leaf production [15] and therefore may be related to the cost of herbivory as metrics for how capable a plant is of tolerating tissue loss. Generally it is expected that plants with high SLA and low LDMC will be more tolerant of grazing[16]. Defense traits (e.g. plant secondary chemistry and leaf toughness) can provide additional insight by articulating a plant’s resistance strategy [17] and are often thought to occur in trade-off with cheap, tolerant leaves although not all results support this trade-off[5, 18, 19].

Altogether, a plant’s response to herbivory, which integrates both its tolerance and resistance features, could be driven by the raw trait values of a species or by the plasticity that a species can exhibit following herbivory[20]. Therefore, we might expect some functional traits to predict whole-plant responses to herbivory, while other traits may vary plastically in response to herbivory. Whether this is the case, and which traits can serve as predictors or responses, remains untested however.

While directly linking explicitly measured traits to plant performance is one way of using traits to predict herbivore impacts[21] it is also possible that multivariate trait complexes shape plant responses[17, 22, 23]. Because resource availability, abiotic stress, and herbivore pressure all contribute to the cost/benefit balance of tolerance and defense, plants with similar historical exposure to herbivory may have evolved similar responses. For example, soil characteristics and herbivore pressure both contribute to habitat specialization in some tropical trees[24].

Ecological theory predicts that at lower latitudes, plants will be subject to greater biomass loss to herbivory from a larger and more rich herbivore community, thereby resulting in stronger herbivore selective pressure on plants in the tropics relative to higher latitudes[25–29]. An association between latitude and plant defense or herbivore load holds for many groups of plants [30, 31] although in some cases the correlation between defenses and latitude may be more reflective of underlying variation in abiotic factors rather than variation in biotic interactions[32–34]. The generality of a latitudinal gradient in herbivory and plant defense has been called into question[35], and a recent analysis found that across taxa most resistance traits were either unrelated to latitude or were greater at higher latitudes[36]. In addition to using functional traits as predictors, in this experiment we sought to test whether variation in latitudinal distribution between taxa, which could capture unmeasured traits or complex syndromes of traits that are affected by the evolutionary history, might relate to their response to herbivory. If a latitudinal gradient in herbivory pressure has produced a greater selective effect of biotic interactions in lower latitudes, we would expect to see a less of a negative response to herbivory in species that have evolved in these high herbivore pressure environments. Alternatively, species that have evolved under high herbivore pressure may have adapted to avoid herbivore damage, and thus, when exposed to a similar damage load will bear a heavier burden then species than have not adapted to limit herbivory.

To assess the response of plants to herbivory in a functional trait context, we utilized a greenhouse approach with simulated defoliation. Common garden or greenhouse studies are useful for assessing plant response to a controlled herbivory stimulus and are preferred when metrics of damage to indicate herbivore pressure are unreliable. Plant responses to simulated defoliation can differ from those elicited by natural herbivory[37–39], not least because herbivory often involves more than just defoliation[40], but growth responses are some of the least sensitive to differences in damage type[41]. More generally, simulated defoliation has great utility despite its shortcomings. It is particularly useful for interrogating simple biotic interactions[42], and it has the advantages of allowing researchers to control for herbivory rate, herbivore preference, and plant defenses across many, phylogenetically distant species[14]. In this study, we used simulated defoliation to focus specifically on the whole plant response to the experience of tissue loss that might come from a leaf-chewing herbivore. Such responses could include a reduction or enhancement in whole plant condition, tolerance (no change), or inducement of a change in phenotype.

This paper seeks to understand variation in plant response to herbivory through a functional traits lens. Specifically, we sought to answer three questions. (1) To what extent do plants respond to simulated defoliation at the whole plant and/or leaf trait level? (2) Do (control) leaf level traits predict whole plant response to simulated defoliation? (3) Does a species’ natural latitudinal distribution predict its response to simulated defoliation?

## Materials and Methods

This study was performed in a greenhouse at Duke University. Plants were exposed to 12 hours of light per day, and received fertilization calibrated to 300 ppm nitrogen with Water Soluble 20-10-20 Peat-Lite Special (Everris NA Inc, Dublin, OH) via watering every two weeks in addition to unfertilized water as needed. All plants were grown in Fafard 4P (Sun Gro Horticulture, Agawam, MA), a standard greenhouse potting mix.

We focused on common old-field species because they exist across a wide latitudinal gradient in the continental United States. Herbivory is known to be important in mediating interspecific competition in old-field communities[43–46]. The species included were selected based on surveys of old-fields[47] and as species frequently documented in a literature review of studies assessing the rate of old-field succession[48]. The species were: *Achillea millefolium*, *Ageratina altissima*, *Andropogon ternarius*, *An*. *virginicus*, *Apocynum cannabinum*, *Asclepias syriaca*, *Coleataenia anceps*, *Dactylis glomerata*, *Daucus carota*, *Festuca arundinacea*, *F*. *rubra*, *Juniperus virginiana*, *Leucanthemum vulgare*, *Liquidambar styraciflua*, *Panicum virgatum*, *Phleum pratense*, *Pinus taeda*, *Poa pratensis*, *Schizachyrium scoparium*, *Securigera varia*, *Solidago altissima*, *So*. *gigantea*, *So*. *juncea*, *So*. *nemoralis*, *Tephrosia virginiana*, and *Verbesina alternifolia*. These species represent a range of life histories (annual to perennial), functional groups (trees, grasses, and forbs), are common in old-fields across eastern North America, and occur at various points during succession (Table 1).

**Table 1:** Summary of species included in this study.

Species	Family	Functional Group	Life History	Latitudinal Midpoint
*Achillea millefolium*	Asteraceae	Forb	Perennial	37.7
*Ageratina altissima*	Asteraceae	Forb	Perennial	36.3
*Andropogon ternarius*	Poaceae	Grass	Perennial	31.725
*Andropogon virginicus*	Poaceae	Grass	Perennial	33.685
*Apocynum cannabinum*	Apocynaceae	Forb	Perennial	38.25
*Asclepias syriaca*	Asclepiadaceae	Forb	Perennial	40.8
*Coleataenia anceps*	Poaceae	Grass	Perennial	33.2
*Dactylis glomerata*	Poaceae	Grass	Perennial	38.25
*Daucus carota*	Apiaceae	Forb	Biennial	37.65
*Festuca arundinacea*	Poaceae	Grass	Perennial	36.8
*Festuca rubra*	Poaceae	Grass	Perennial	41.15
*Juniperus virginiana*	Cupressaceae	Tree	Perennial	35
*Leucanthemum vulgare*	Asteraceae	Forb	Perennial	38.8
*Liquidambar styraciflua*	Hamamelidaceae	Tree	Perennial	34.05
*Panicum virgatum*	Poaceae	Grass	Perennial	35.2
*Phleum pratense*	Poaceae	Grass	Perennial	41.05
*Pinus taeda*	Pinaceae	Tree	Perennial	33.85
*Poa pratensis*	Poaceae	Grass	Perennial	39.55
*Schizachyrium scoparium*	Poaceae	Grass	Perennial	36.05
*Securigera varia*	Fabaceae	Forb	Perennial	38.7
*Solidago altissima*	Asteraceae	Forb	Perennial	38.25
*Solidago gigantea*	Asteraceae	Forb	Perennial	38.85
*Solidago juncea*	Asteraceae	Forb	Perennial	40.8
*Solidago nemoralis*	Asteraceae	Forb	Perennial	39.65
*Tephrosia virginiana*	Fabaceae	Forb	Perennial	35.3
*Verbesina alternifoli*	Asteraceae	Forb	Perennial	36.1

Individuals were grown from seed that was purchased commercially (*So*. *gigantea* and *T*. *virginiana*—Prairie Moon Nursery, Winona, MN; all others—Ernst Conservation Seeds, Meadville, PA) except for *So*. *altissima*, which was collected in Durham, NC. After four weeks in a greenhouse germination room, ten individuals were transplanted into 7.65 liter tree pots and arranged in in a randomized block design. After 14 weeks growth in the tree pots, all individuals were harvested.

### Simulated Defoliation

To examine the effects of herbivory on expression of functional traits, five individuals went unmanipulated and five individuals of each species were exposed to a simulated defoliation regime. These individuals were randomly assigned to the herbivory treatment when transplanted into the tree pots and were allowed to grow unmanipulated for 5 weeks. Then on June 11 2013, 10% of the leaf tissue was removed from each individual. While this value of simulated herbivory was relatively low for greenhouse studies[49–54], it is representative of *in situ* herbivory rates and thus provides more realistic response rates. The area of tissue removal was selected as an average based on field surveys of herbivory damage on *Solidago altissima* from Florida to New York (Joshua Lynn, unpublished data). Since herbivory is expected to vary across the latitudinal gradient and as such seeds sourced from different locations would have been subject to different herbivory regimes, we chose a lower value of simulated herbivory to more closely hew to expected damage rates for all taxa. For the grass species (*An*. *ternarius*, *An*. *virginicus*, *Dac*. *glomerata*, *F*. *arundinacea*, *F*. *rubra*, *Co*. *anceps*, *Pa*. *virgatum*, *Ph*. *pratense*, *Po*. *pratensis*, *Sc*. *scoparium*), this entailed cutting the top 10% of each leaf blade. For the herbaceous species (*Ac*. *millefolium*, *Ag*. *altissima*, *Ap*. *cannabinum*, *Asc*. *syriaca*, *Dau*. *carota*, *Le*. *vulgare*, *Se*. *varia*, *So*. *altissima*, *So*. *gigantea*, *So*. *juncea*, *So*. *nemoralis*, *T*. *virginiana*, *V*. *alternifolia*), and the deciduous tree (*Li*. *styraciflua*), this entailed removing 10% of the area from each leaf with a 1mm paper hole puncher or scissors. Leaf area was calculated with a sizing tool marked with nested squares and rectangles of various size classes[55]. For the coniferous trees (*J*. *virginiana* and *Pi*. *taeda*), the simulated defoliation regime entailed removing 10% of the needles. This procedure was repeated on all new leaves on July 15 2013, for a total of two simulated defoliation events over the span of 1 month.

### Trait Measurements

The following traits were measured: specific leaf area (SLA); leaf dry matter content (LDMC); ash content; and leaf toughness[17, 56]. SLA and LDMC were measured as constituents of the leaf economic spectrum that represent a general trade-off between leaves with high construction cost, low carbon acquisition, and long life span (low SLA and high LDMC) versus relatively cheap, productive, and short lived leaves (high SLA and low LDMC)[57, 58]. Leaf toughness is an important form of resistance against herbivores[59–62]. Ash content can be used as a proxy for resistance traits such as silica-based phytoliths and calcium oxalates[36]. We also measured final height, total leaf area, and total aboveground biomass as performance variables at the end of 14 weeks. All data can be found in Supporting Information.

To generate an estimate of where each species tends to be found along the latitudinal gradient, we used county level range maps from the Biota of North America Program (bonap.org), and determined the midpoint between the most southern and northern points at which a species was present (Table 1). Because county level range maps only exist for the United States, species that extend their range north into Canada have a southern bias to their latitudinal estimate, which will result overall in a conservative estimate of latitudinal variation. We also calculated another measure of species’ latitude, the cover-weighted mean latitude from data presented in [63]. This alternate measure was highly correlated with the mid-point of the range (p<0.001, R^2^ = 0.65; S1 Fig) and since cover-weighted estimates were not available for all of the species in our study, we only used the mid-point estimate in our analyses. Seeds were sourced commercially, so their specific latitude of origin could not be determined. State of origin was significantly correlated with midpoint latitude (P<0.001, R^2^ = 0.63), however, and so the rank distribution of origin and that from the distribution analysis were similar. Using midpoint latitude would likely have resulted in a weaker relationship with plant responses than the actual source latitude since it does not capture intraspecific variation in herbivory response. However, it serves as an estimate of differences between species in evolutionary history of exposure to herbivory.

### Analyses

We first tested for a phylogenetic signal in the functional trait and latitude data. We generated a phylogeny for the species in the experiment in Phylomatic v3 (www.phylodiversity.net/phlyomatic/) using the Zanne et al. stored tree[64]. We then calculated phylogenetic independent contrasts for each trait using the “phylosignal” function in the R package picante[65]. After performing Bonferroni multiple-hypothesis correction, we observed no significant phylogenetic signal in any of the traits. Therefore, we did not include phylogenetic corrections in any of the downstream analyses of treatment effects.

We first assessed whether herbivory affected performance (height, aboveground biomass, and total leaf area) and whether these effects differed across species by using linear mixed effects models. We entered herbivory treatment and species (with their interactions) into the model as fixed effects, and used block as a random effect. P-values were obtained by likelihood ratio tests of the full model with the effect in question against the model without the effect in question, and they underwent Bonferroni multiple-hypothesis correction. We performed a similar analysis to determine whether herbivory affects leaf level traits using SLA, LDMC, ash content, and leaf toughness as response variables.

To explore whether the response to simulated defoliation was predictable based on plant functional traits or latitude, we paired each control plant with one experiencing simulated defoliation within each species, and computed the log ratio of treated to control plants for their height, aboveground biomass, and total leaf area. We then regressed these log ratios against each of the trait values of the control plant in each pair and against the mid-point latitude at which they are found. To ensure that the observed result was not the result of block-level effect we used a bootstrapping procedure where we repeated the random pairing of control and treatment plants within species and recalculated the regression values. This process was repeated 10,000 times and we looked at the mean effect across all iterations. To adjust for multiple-hypothesis testing here, we report the 99% confidence intervals of the slopes, intercepts, and r^2^ values. Regressions where the slope 99% confidence interval did not include 0 (i.e. all values were positive or negative) were considered significant relationships.

Finally, to test whether latitude was capturing features of functional trait variability, we conducted linear regressions of control leaf traits against mid-point latitude and performed Bonferroni multiple-hypothesis corrections. All statistics were carried out in R (version 3.1.1; www.r-project.org) and mixed effects models were computed using the lme4 package[66].

## Results

**Fig 1:**
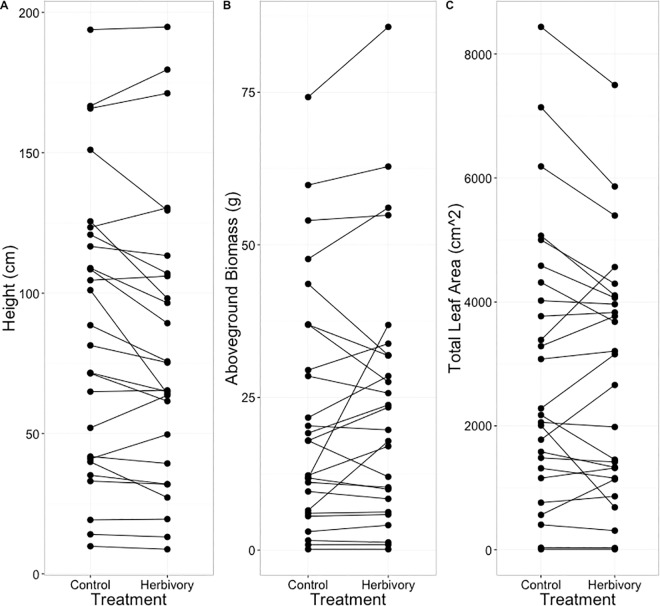
Whole plant performance responses to simulated defoliation. Average (A) height, (B) aboveground biomass, and (C) total leaf area for each species treatment group. Positive, negative, and non-significant differences between herbivory and control groups were observed for each performance variable, but there were no significant effects of treatment.

None of the whole plant traits (height, aboveground biomass, total leaf area) were significantly affected by defoliation, either as a main effect or in interaction with species identity (P>0.05; Fig 1). Furthermore, none of the best models with leaf level traits as response variables contained simulated defoliation treatment as a variable.

In the models exploring whether the response to simulated defoliation was predictable using functional trait data (Table 2), SLA had a consistently significant, but small, negative correlation with the log response ratio for all performance variables. LDMC had a significant, positive correlation with the log response ratio for biomass and total leaf area but had no relationship with height. Toughness was negatively correlated with height but uncorrelated with biomass and total leaf area. Leaf ash content was uncorrelated with any of the whole-plant performance variables.

**Table 2:** Leaf-level traits and latitude can predict whole-plant responses to herbivory.

	Slope mean	Slope 99% interval	Intercept mean	Intercept 99% interval	r^2^ mean	r^2^ 99% interval
Height ~ Latitude	0.062	[-0.047, 0.175]	**-0.062**	[-0.076, -0.040]	0.004	[0.000, 0.022]
Height ~ SLA	**-0.183**	[-0.394, -0.059]	**-0.069**	[-0.090, -0.042]	0.030	[0.003, 0.102]
Height ~ LDMC	-0.056	[-0.177, 0.056]	**-0.072**	[-0.089, -0.048]	0.004	[0.000, 0.025]
Height ~ Toughness	**-0.134**	[-0.251, -0.023]	**-0.196**	[-0.222, -0.161]	0.017	[0.000, 0.052]
Height ~ Ash Content	-0.105	[-0.330, 0.122]	**-0.066**	[-0.085, -0.041]	0.007	[0.000, 0.036]
			** **			
Biomass ~ Latitude	**-0.499**	[-0.742, -0.243]	**0.064**	[0.022, 0.101]	0.048	[0.011, 0.099]
Biomass ~ SLA	**-0.328**	[-0.688, -0.073]	0.038	[-0.018, 0.085]	0.025	[0.001, 0.085]
Biomass ~ LDMC	**0.389**	[0.004, 0.667]	**0.035**	[-0.005, 0.072]	0.035	[0.000, 0.091]
Biomass ~ Toughness	-0.004	[-0.259, 0.249]	**-0.200**	[-0.268, -0.136]	0.002	[0.000, 0.017]
Biomass ~ Ash Content	0.022	[-0.491, 0.508]	**0.056**	[0.000, 0.110]	0.005	[0.000, 0.022]
			** **			
Leaf Area ~ Latitude	**-0.529**	[-0.765, -0.288]	-0.030	[-0.076, 0.015]	0.047	[0.014, 0.093]
Leaf Area ~ SLA	**-0.554**	[-0.984, -0.244]	**-0.065**	[-0.125, -0.008]	0.061	[0.012, 0.149]
Leaf Area ~ LDMC	**0.414**	[0.066, 0.693]	**-0.072**	[-0.121, -0.029]	0.035	[0.001, 0.088]
Leaf Area ~ Toughness	-0.040	[-0.313, 0.22]	**-0.416**	[-0.475, -0.363]	0.003	[0.000, 0.022]
Leaf Area ~ Ash Content	-0.151	[-0.819, 0.458]	-0.047	[-0.110, 0.015]	0.009	[0.000, 0.049]

Regression parameters and r^2^ values for each model testing whether the response to herbivory was predictable based on traits and latitude variables. 99% intervals correspond to the middle 99% of values across the 10,000 bootstrap iterations. Significant slope and intercept terms are indicated in bold.

In the models exploring whether the response to simulated defoliation was predictable using latitudinal data (Table 2), we found that latitudinal midpoint exhibited a negative correlation with the log response ratio for biomass and total leaf area. This indicates that plants with a higher midpoint latitude (from farther north) had lower log ratio and thus treated individuals were more negatively impacted. Midpoint latitude had the largest average explanatory value (R^2^ = 0.048) of any of the traits associated with biomass.

**Fig 2:**
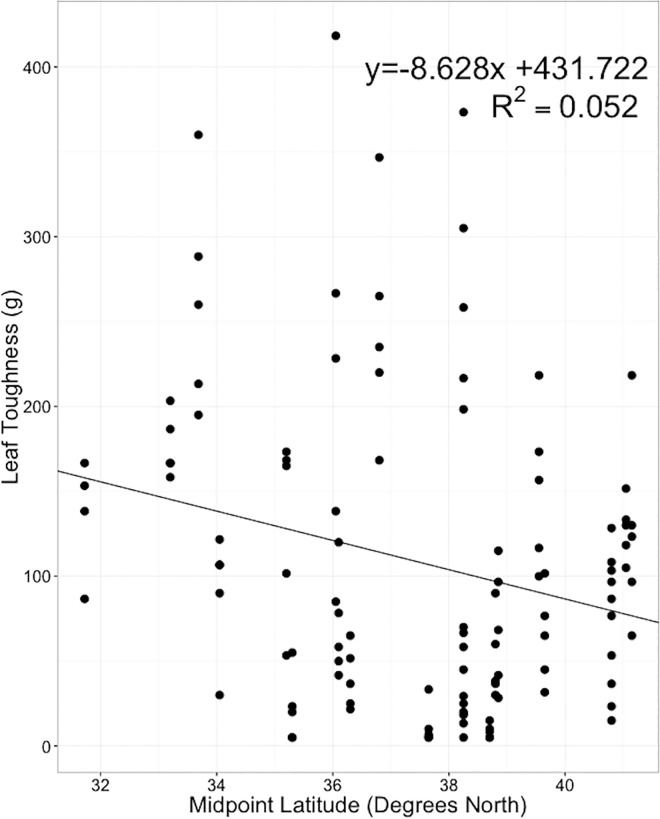
Correlation between latitude and leaf toughness. Leaf toughness of untreated plants is greater (p = 0.002) in species with more southern midpoint range.

Most leaf level traits were not associated with latitude. The exception was toughness, which was exhibited a negative relationship with latitude indicating that more southern plants had greater toughness (P = 0.002; Fig 2).

## Discussion

In our study, low, but ecologically relevant, levels of simulated defoliation had overall limited main effects on plant performance. But the consistent observed relationships between traits and responses to herbivory provide new insights about what may underlie some of the variability in response. Herbivory is a complex phenomenon that can affect plants through many pathways, and functional traits may contribute to assessing and predicting the relevant responses in a given interaction context.

Of the three whole-plant performance variables, none were significantly affected by herbivory. Many of the induced effects of herbivory respond to cues other than simply tissue loss (e.g. chemical cues in saliva; [41]), which may explain why few herbivory treatment effects were observed in our study. Similarly, we expect little light or nutrient limitation in the greenhouse, which may dampen any effects of herbivory. While occasional herbivore outbreaks can lead to significant defoliation, in old-field systems, most herbivores are relatively rare most of the time[67], leading most plants to experience chronic low-levels of tissue loss as were applied in this experiment. Additionally, while SLA and LMDC are highly plastic traits in response to environmental drivers[68–70], and herbivory is well known to induce a broad array of changes in plants, we found no evidence that simulated defoliation significantly affected any of the leaf-level traits. It is possible that this lack of a response was due the relatively low levels of defoliation.

While the overall impact of simulated defoliation was primarily not significant, we still observed variation in the whole-plant performance variables. The analysis of response effects produced significant correlations but with generally low R^2^ values, indicating that while there is a functional relationship between responses to defoliation and plant traits, traits cannot predict all of the variation in response. While not strong predictors of plant responses to herbivory, the directions of the responses that we observed are consistent mechanistic links between plant traits and plant performance. The correlations between the performance response and SLA or LDMC indicate that plants with high SLA and low LDMC incur higher herbivory costs per unit leaf loss. This is in accordance with the expectation that in high light conditions plants with low SLA are expected to outperform those with high SLA, even if they receive greater herbivore damage[71, 72]. Such a pattern may reflect a high nitrogen cost of rebuilding leaves for plants with high SLA and low LDMC. Leaves with high SLA and low LDMC are generally considered to be cheaper leaves[58]. This definition primarily captures carbon costs, and in the competition-free setting of the greenhouse, carbon fixation is not expected to be limiting. If nitrogen were more limiting, plants with high SLA and low LDMC would be expected to be more affected by tissue loss than those with low SLA and high LDMC. In this experiment, however, plants were unlikely to experience extremely nitrogen-limiting conditions, so further research will be necessary to identify if nitrogen limitation is in play. A more general explanation could be that the sensitivity to herbivory is related to growth rate as a reflection of adaptation for high- or low- nutrient environments (Resource Availability Hypothesis)[73], but this relationship may not be apparent at the level of intra-specific variation.

In contrast, toughness is generally reflective of an increase in the cell wall: cytoplasm ratio which can also be considered a crude fiber: crude protein ratio[60], and so is not necessarily nitrogen intense. The observed relationship between high toughness and height response therefore indicates that further limiting elements beyond carbon and nitrogen may need to be considered to model whole-plant responses to herbivory. Generally, leaf toughness is negatively correlated with growth rate[74], and herbivory may enhance that effect.

The relationship between latitudinal midpoint and biomass and total leaf area indicates that more northern taxa were more negatively impacted by herbivory. If there is a latitudinal gradient in herbivory pressure, with southern plants receiving more herbivory, then it is to be expected that when exposed to a controlled amount of herbivory, southern plants would be more tolerant or better defended. Our finding that toughness was negatively correlated with latitude corroborates this hypothesis. However, this result is in contrast to a recent analysis covering a much larger geographic range that measured traits *in situ*[36]. This difference accentuates how much context can matter in analyzing biogeographic variability.

It is important to recognize that because our seeds were sourced commercially, the latitudinal midpoint of a species reflects its broader evolutionary history of exposure to herbivory, and not more immediate effects such as maternal or epigenetic effects. Tests with multiple populations of species collected from different latitudes would represent a stronger test of the hypothesis that latitude of origin drives species’ responses to herbivory. However, the sourcing patterns do recapitulate natural variation in distributions, and the fact that we observed a relationship between historical range even with the impacts weakened by sourcing from a small number of locales suggests that this pattern is worth further investigation.

There is little consistent evidence for a latitudinal gradient in herbivory[35], but in studies of more limited groups of plants there may be more support[11, 75]. Furthermore, evidence has been found for variation in resistance and tolerance[8] as well as inducibility of defense[30, 33] when analyzing single clades from multiple locales. Our study focused on a suite of species that are found in old fields across eastern North America, for which, while occurring in communities undergoing similar successional trajectories, site level variation in edaphic factors and traits is known to exist[47, 48]. The role herbivory may play in old field successional change remains unclear, but if the patterns of response to herbivory along latitude of origin seen here hold in the field, it would suggest that herbivory might contribute to the slower rate of succession observed in higher latitudes.

While simulated defoliation had a fairly minimal overall impact in our study, we identified a number of traits that help predict responses to herbivory. These results indicate the promise of using traits to predict responses to herbivory. In particular, this is among the first studies to consider the role of individual level trait responses in predicting whole plant herbivory responses. Future studies should specifically focus on approaches that can disentangle the mechanism underlying variation in responses and explain the variation in plant response that was unaccounted for in our models. One possible mechanism of the patterns we observed that requires future research is the importance of nitrogen versus carbon costs to defense and tolerance. Additional focus on the relative impact of herbivory and traits on different performance variables (i.e. height vs. leaf area) will also be necessary to disentangle the ecological and evolutionary implications of herbivory.

## Supporting Information

S1 Fig Significant correlation between midpoint latitude and cover-weighted mean latitude. Midpoint Latitude and cover-weighted mean latitude (as calculated from Siefert et al. 2014) are highly correlated (p<0.0001, R2 = 0.65) although the slope of this relationship (solid line) does differ from 1 (dashed line). Midpoint latitude was used for all analyses because, while it does not adjust for abundance, it was available for all species.
(TIF)

S1 Dataset Contains all trait and distribution data used in these analyses. Raw (N = 3–6) and average values for traits are included.
(CSV)
