# Supplementary figures and images for: Variation in Plant Response to Herbivory Underscored by Functional Traits
Source: PLoS One. 2016 Dec 9;11(12):e0166714. doi: 10.1371/journal.pone.0166714 (PMC5147848; doi:10.1371/journal.pone.0166714)

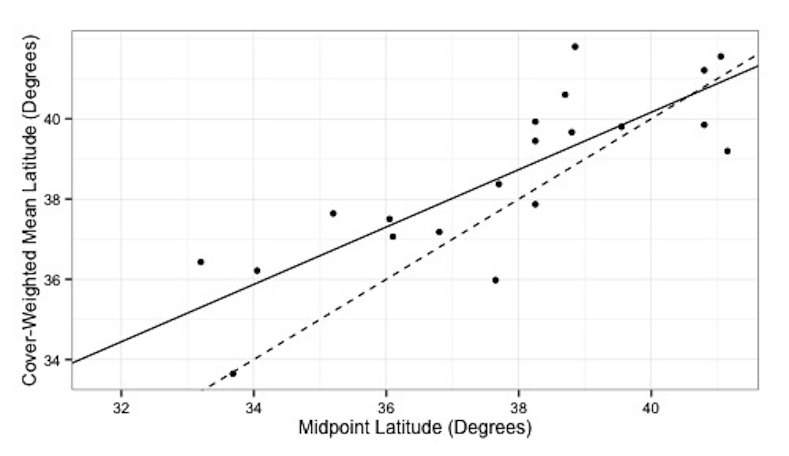

Supplement: S1 Fig — Midpoint Latitude and cover-weighted mean latitude (as calculated from Siefert et al. 2014) are highly correlated (p<0.0001, R2 = 0.65) although the slope of this relationship (solid line) does differ from 1 (dashed line). Midpoint latitude was used for all analyses because, while it does not adjust for abundance, it was available for all species. (TIF) [file pone.0166714.s001.tif]
